# Impacts from air pollution on respiratory disease outcomes: a meta-analysis

**DOI:** 10.3389/fpubh.2024.1417450

**Published:** 2024-10-09

**Authors:** Jason G. Su, Shadi Aslebagh, Eahsan Shahriary, Meredith Barrett, John Randolph Balmes

**Affiliations:** ^1^School of Public Health, University of California, Berkeley, CA, United States; ^2^ResMed, San Diego, CA, United States; ^3^School of Medicine, University of California, San Francisco, CA, United States

**Keywords:** air pollutants, trace metals, respiratory disease, meta-analysis, public health

## Abstract

**Introduction:**

Air pollution is widely acknowledged as a significant factor in respiratory outcomes, including coughing, wheezing, emergency department (ED) visits, and even death. Although several literature reviews have confirmed the association between air pollution and respiratory outcomes, they often did not standardize associations across different studies and overlooked other increasingly impactful pollutants such as trace metals. Recognizing the importance of consistent comparison and emissions of non-exhaust particles from road traffic, this study aims to comprehensively evaluate the standardized effects of various criteria pollutants and trace metals on respiratory health.

**Methods:**

We conducted a comprehensive meta-analysis of peer-reviewed journal articles on air pollution and respiratory outcomes published between 1 January 2000, and 1 June 2024. The study included children (age < 18 years), adults (age ≥ 18 years), and all age groups exposed to criteria pollutants established by the US Environmental Protection Agency National Ambient Air Quality Standards and over 10 trace metals. Using databases, such as PubMed, MEDLINE, Web of Science Core Collection, and Google Scholar, we identified 579 relevant articles. After rigorous screening and quality assessment using the Newcastle-Ottawa Scale, 50 high-quality studies were included. We converted various reported outcomes (e.g., odds ratios, relative risk, and percent increase) to a standardized odds ratio (OR) for comparability and performed meta-analyses using R 4.4.0 and related packages, ensuring the robustness of our findings.

**Results:**

Our meta-analysis indicated significant associations between air pollutants and respiratory outcomes. For particulate matter with diameter ≤ 2.5 μm (PM_2.5_), the overall ORs for children, adults, and combined age groups were 1.31, 1.10, and 1.26, respectively, indicating a consistent positive association. Similar positive associations were observed for particulate matter with diameter ≤ 10 μm (PM_10_) and other pollutants, with children showing higher susceptibility than adults. The analysis of trace metals also showed significant associations; however, these findings require cautious interpretation due to the small number of studies.

**Conclusion:**

Our study supports associations between air pollutants, including non-exhaust trace metals, and respiratory outcomes across different age groups. The findings underscore the need for stringent environmental health policies and further research, especially in regions with higher pollution levels. The future studies should consider long-term and short-term exposures separately and include diverse populations to improve the accuracy and generalizability of the results.

## 1 Introduction

It is well established that air pollution is associated with respiratory disease outcomes, such as coughing (1–3), wheezing (4–7), emergency department (ED) visits (8, 9), and even death (10–14). Some literature reviews (3, 8, 13, 15–17) have been conducted on those associations, and they essentially confirmed the associations of air pollution exposure with respiratory disease outcomes. However, these literature reviews did not assess many pollutants, such as trace metals that can be considered toxic air contaminants, nor did they assess associations found in different studies on the same scale.

Past analyses typically used different interquartile range increments in assessing an association, making the impact size challenging to compare across studies. Some studies used odds ratios (OR) of an outcome occurring (2–7) while others used percent increase (1, 14) or relative risk (RR) in assessing the association of air pollution exposure with respiratory outcomes (8, 15).

It is well recognized that on-road vehicle traffic contributes significantly to air pollution locally and regionally. On-road vehicles contribute to ambient air pollution from engine emissions, automobile brakes, tire wear, and dust from road surfaces (18). Non-exhaust particles are generated from brakes, tires, clutch, and road surface sources. In areas with higher traffic density and more braking frequency, brake wear is a significant particulate matter (PM) contributor among all non-exhaust pollutant sources (19). Another PM source is road surface wear. Furthermore, particles that already exist in the form of debris at the roadside get resuspended due to the turbulence created by on-road vehicular traffic and contribute to ambient PM generation. Previous studies show over half of the mean PM_2.5_ (PM with diameter < 2.5 μm) is a cumulative result of on-road motor vehicles (42%) and road dust (12%) (20).

Due to the increasing implementation of tailpipe emission regulations, non-exhaust emissions from tire and brake wear have become increasingly important. The fraction of PM_2.5_ from non-exhaust sources is expected to increase as electric vehicles replace internal combustion vehicles. Studies have shown that even with zero exhaust emissions from on-road vehicles, traffic will continue contributing to PM_10_ (PM with diameter < 10 μm) and PM_2.5_ through non-exhaust emissions (19). Some studies have shown that air pollution from tire and brake wear increases asthma morbidity and mortality risks (10, 21).

Air pollution’s significant health impacts align with the sustainable development goals (SDGs). SDG 3 aims to promote the health and well-being of those directly affected by air pollution contributing to respiratory diseases, such as asthma and lung cancer. The World Health Organization (WHO) highlights that air pollution causes millions of premature deaths annually (22). Furthermore, SDG 12 targets reducing emissions of hazardous chemicals, including criteria pollutants such as PM and toxic trace metals such as lead and mercury, which have severe health impacts. Achieving these goals necessitates stronger air quality regulations, improved monitoring, and the adoption of cleaner technologies across industries and transportation sectors (23). By assessing the effects of criteria pollutants and trace metals on respiratory health through standardized meta-analysis, we contribute to broader efforts aimed at mitigating air pollution’s adverse effects and advancing global health and environmental sustainability goals.

Therefore, it is essential to understand the association between criteria pollutants and trace metals and respiratory disease, where such knowledge is currently limited. In this study, we performed a meta-analysis to assess the effect sizes of the associations between exposures to criteria pollutants, including nitrogen dioxide (NO_2_), PM_2.5,_ PM_10_, ozone (O_3_), sulfur dioxide (SO_2_), and more than 10 trace metals that can be considered toxic air contaminants and respiratory disease outcomes to shed light on comparative risk.

## 2 Materials and methods

### 2.1 Selection of articles for review and data extraction

We conducted a systematic literature review using peer-reviewed journal articles, to expand the literature cited in the background section of this study on the impacts of on-road vehicle emissions, including on-road non-exhaust pollutants, on subacute respiratory disease symptoms. The following interconnected steps were used to complete the review:

- Determine inclusion criteria that will include the following:
  - Study population of children (age < 18 years), adults (age ≥ 18 years), and all age groups.
  - Study intervention for individuals (1) exposed to air pollution, including NO_2_, PM_2.5_, PM_10_, O_3_, and SO_2_, and (2) more than 10 trace metals are considered toxic air contaminants (including aluminum, iron, magnesium, sulfur, nickel, vanadium, chromium, arsenic, manganese, barium, copper, antimony, zinc, and lead).
  - Study outcomes for respiratory disease in asthma and chronic obstructive pulmonary disease (COPD), such as coughing, wheezing, shortness of breath, ED visits, hospitalizations, exacerbations and mortality, respiratory infections, and lung cancer.
- Identify the publication’s characteristics for studies:
  - Published in peer-reviewed journals;
  - Published between 1 January 2000 and 1 June 2024;
  - Written in English.
- Select the proper search databases and engines, including
  - PubMed;
  - Medline;
  - Web of Science Core Collection; and
  - Google Scholar.
- Decide the search terms and selection process.

Table 1 summarizes pollutant categories and search criteria considered for this literature review.

**Table 1:** Literature review categories and search terms.

Category	Search terms
Air pollutants	PM_2.5_, PM_10_, NO_2_, O_3_, SO_2_, and trace metals
Disease	Asthma, COPD, and respiratory disease
Years of publication	From 1 January 2000 to 1 June 2024
Publication type	Peer-reviewed journals
Publication language	English

COPD, chronic obstructive pulmonary disease; PM, particulate matter.

The following steps were used to select scientific publications for the literature review:

1. Use one term from each category and combine them together (+) to create integrated search terms using the search databases and engines listed above.
2. Merge together the selected publications and remove the duplicates.
3. Obtain abstracts for the remaining publications selected from Step 2, screen and remove the publications that are not related to the research topic.
4. Obtain full text for the remaining publications selected from Step 3, screen and remove the publications that are not related to the topic.

We identified 579 articles considering the search criteria given in Table 1. After screening titles, 197 articles were selected for an abstract search. We evaluated the abstract of each article and selected 114 articles for full-text screening. After checking the risk of bias using the Newcastle–Ottawa scale (NOS) (24), 50 articles were rated as good or high quality (6–9 score; Supplementary Table S37) and included in the final review.

Two independent researchers reviewed the publications and extracted the information. The data of first author, year, location, air pollutant, study design, sample size, study group, and outcome were collected. We investigated the effect of increased exposure to different criteria pollutants, including PM_2.5_, PM_10_, NO_2_, O_3_, SO_2_, and trace metals, including aluminum, iron, magnesium, sulfur, nickel, vanadium, chromium, arsenic, manganese, barium, copper, antimony, zinc, and lead. For health outcome measures, we included coughing, wheezing, shortness of breath, ED visits, hospitalizations, exacerbations, and mortality for respiratory diseases, including asthma, chronic obstructive pulmonary disease (COPD), respiratory infections, and lung cancer.

### 2.2 Conversion of outcomes to OR

Not all studies in this systematic review reported the results on the same scale. Estimated outcomes associated with increased exposure to air pollutants are reported in terms of odds ratio (OR), relative risk (RR) or percentage increase (%), and 95% confidence intervals (CI). We standardized all reported results by converting them to odds ratios (ORs) with corresponding confidence intervals (CIs) to facilitate comparison across different studies. By aggregating these standardized effects, we determined the overall impact of each pollutant on respiratory disease outcomes. Since the exposure interval differs for each metal, we did not standardize the reported OR/RR and 95% CI, and they were presented in the same way as reported in the corresponding studies.

To convert the results reported in percentage increase to OR, we exponentiated the reported results and lower/upper CI values from the corresponding studies, as presented in the following set of formulas:


OR=exp(%increase)

$${\mathrm{CI}}_{\mathrm{lower}}=\exp\;\left(\%{\mathrm{CI}}_{\mathrm{lower}}\right)$$

$${\mathrm{CI}}_{\mathrm{upper}}=\exp\;\left(\%{\mathrm{CI}}_{\mathrm{upper}}\right)$$

Also, to convert the results reported in RR to OR, the following conversion was used (25):


$$OR=\left(1-{\mathrm{risk}}_{0}\right)\times \frac{\mathrm{RR}}{1-{\mathrm{risk}}_{0}\times \mathrm{RR}}$$

Where risk_0_ is the risk of having a positive outcome in the control or unexposed group; similarly, the associated lower and upper C.I.s can be calculated as follows:


$${\mathrm{CI}}_{\mathrm{lower}}=\left(1-{\mathrm{risk}}_{0}\right)\times \frac{{\mathrm{RR}}_{\mathrm{lower}}}{1-{\mathrm{risk}}_{0}\times {\mathrm{RR}}_{\mathrm{lower}}}$$

$${\mathrm{CI}}_{\mathrm{upper}}=\left(1-{\mathrm{risk}}_{0}\right)\times \frac{{\mathrm{RR}}_{\mathrm{upper}}}{1-{\mathrm{risk}}_{0}\times {\mathrm{RR}}_{\mathrm{upper}}}$$

### 2.3 Standardization of exposure increase ranges

Furthermore, not all studies used the same exposure interval to assess the association with respiratory diseases. We standardized all the reported results so that the results were comparable between various studies. To make the results similar between different studies, we standardized the ORs through the following conversions:


$$\mathrm{OR}\left(x_{s}\right)=\mathrm{OR}{\left(x_{g}\right)}^{\frac{x_{s}}{x_{g}}}$$

where OR(*x*_s_) is the standardized OR for pollutant *x* when its exposure interval is set at *x*_s_; *x*_g_ and OR(*x*_g_) represent the standardized exposure interval and associated OR. Based on the potential exposure levels from current studies, we used interquartile ranges of 10 ppb, 10 μg m^−3^, 10 μg m^−3^, 10 ppb, and 30 ppb, respectively, for NO_2_, PM_2.5_, PM_10_, SO_2_, and O_3_ in standardizing exposure level.

### 2.4 Statistical analysis

We excluded the studies deemed significant outliers, such as OR greater than 4.0. After standardizing the reported results from studies, effect pooled analysis was performed for each pollutant in three different age categories, including children ( < 18 years), adults ( ≥ 18 years), and all ages. The R Foundation for Statistical Computing (R 4.4.0) and “outliers,” “metafor” and “meta” packages were used for meta-analysis of the effects, separately, for children, adults, and all ages, for NO_2_, PM_2.5_, PM_10_, SO_2_, O_3_, and trace metals. Studies with mixed ages were evaluated separately from those studies focused on just children or adults to avoid the potentially repeated counting of children and adults in the all age category (i.e., we did not pool the effects from children, adults, and all age groups to form a category of all subjects). We conducted a sensitivity analysis to explore the robustness of the meta-analysis results. In this process, we recalculated the overall homogeneity and effect size by systematically removing one study at a time. The heterogeneity was tested using Cochran’s Q test (26, 27) and the *I*^2^ value (28, 29). When *I*^2^ ≤ 50%, the studies were considered homoscedastic, and the fixed effect model of the meta-analysis was used; when *I*^2^ > 50%, the studies were deemed heteroscedastic, and the random effect model of the meta-analysis was used. Publication bias was tested using a regression-based Egger test and funnel plot (30). The significance level was *p* < 0.05.

## 3 Results

### 3.1 Search results and study descriptions

**Figure 1:**
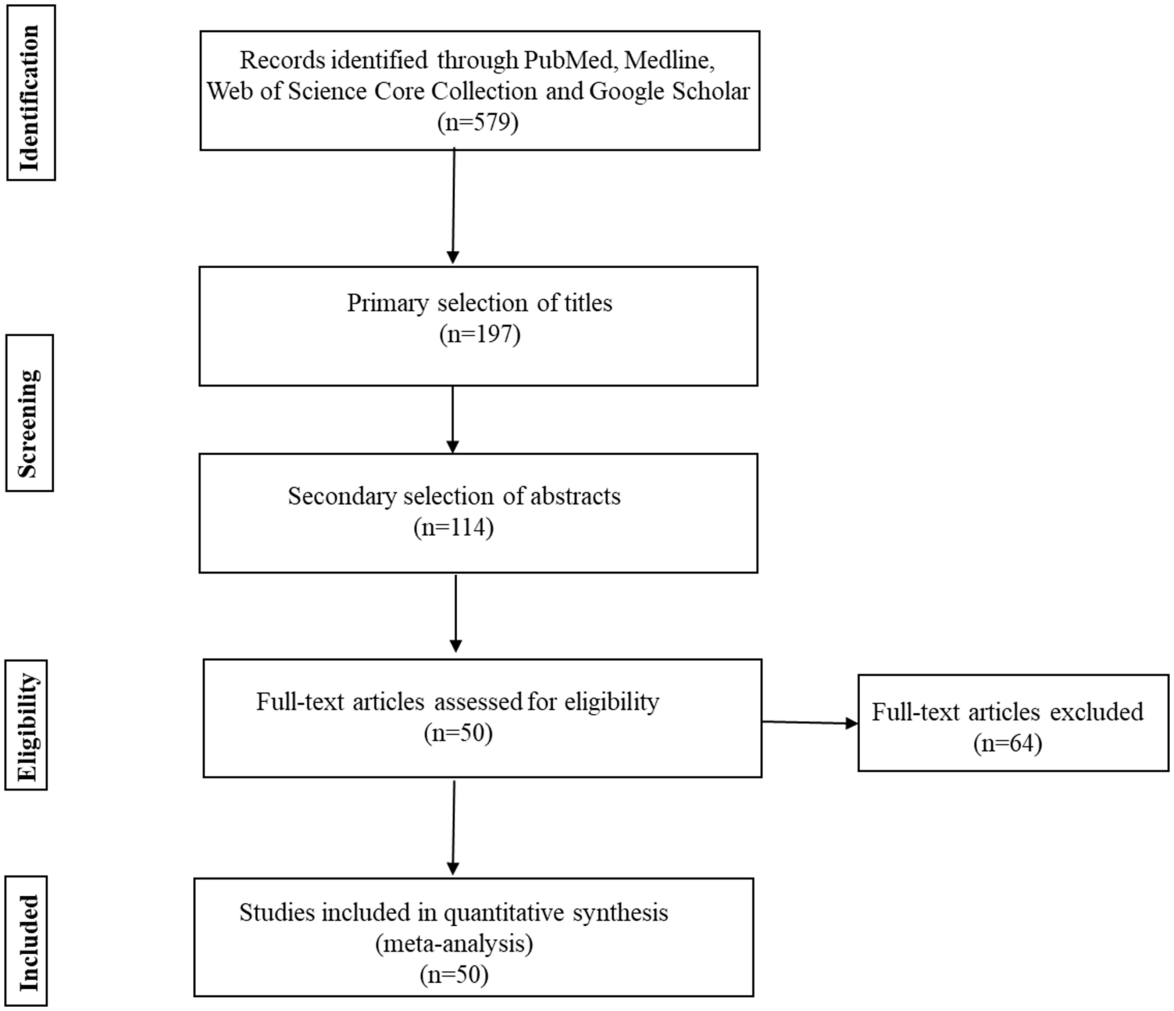
Flow diagram summarizing study selection.

We identified 579 articles considering the search criteria through databases. After screening titles, 197 articles were selected for an abstract search. We screened the abstract of each article and selected 114 articles for full-text assessment. After the full-text assessment, 50 articles were included in the final review and quantitative synthesis (Figure 1).

The studies were conducted in multiple countries as follows: Australia (1), Belgium (1), Canada (3), China (12), multiple countries in Europe (2), Germany (1), India (1), Iran (2), Italy (4), Israel (1), Japan (3), Mexico (1), The Netherlands (1), Spain (1), South Korea (1), Taiwan (2), Thailand (1), Turkey (1), United Kingdom (1), and United States (12). Among 50 studies,13 studies included children (<18 years), 19 studies included adults (≥18 years), 16 studies included all ages, 2 studies included children (<18 years) and adults (≥18 years), and 1 study included children (<18 years), adults (≥18 years), and all ages (Table 2).

**Table 2:** Characteristics of 50 studies included in the meta-analysis.

First author	Year	Location	Air pollutant	Study design	Sample size	Study group	Outcome
De Marco (37)	2002	Italy	NO_2_	Cross-sectional survey	18,873	Adults	Asthma attack, chest tightness, wheezing
Sunyer (38)	2002	Spain	NO_2_	Case-crossover design	1,078	All	Mortality due to asthma
Gent (39)	2003	USA	O_3_	Prospective study	271	Children	Chest tightness(1 h), Shortness of breath (1 h), Chest tightness(8-h), Shortness of breath (8 h)
Mar (2)	2004	USA	PM_2.5_, PM_10_	Unknown	25	children, adults	Trouble breathing, coughing, sputum production, wheezing
Migliaretti (40)	2004	Italy	NO_2_	Case–control study	1,060	Children	Asthma hospitalizations
Analitis (41)	2006	Europe	PM_10_	Unknown	Unknown	All	Respiratory mortality
Brauer (5)	2007	Netherlands	PM_2.5_	PIAMA prospective birth cohort study	~ 4,000	Children	Wheezing, doctor-diagnosed asthma, ear/nose/throat infections, flu
Smargiassi (42)	2009	Canada	SO_2_	Time-stratified case-crossover	3,469	Children	Asthma emergency department visits, asthma hospitalization
Kan (43)	2010	Thailand, China	SO_2_	Short-term association study	Unknown	Adults	Respiratory mortality
Silverman (44)	2010	USA	O_3_	Unknown	75,383	Adults	Asthma HA
Weinmayr (3)	2010	Germany/Italy	PM_10_, NO_2_	Meta-analysis	36	Children	Asthma symptoms, coughing, asthma symptoms
Katanoda (45)	2011	Japan	PM_2.5_, NO_2_, SO_2_	Prospective cohort study	63,520	All	Mortality due to lung cancer and respiratory diseases
Takenoue (6)	2012	Japan	NO_2_	Meta-analysis	12	Children	Asthma development, wheezing
Belanger (4)	2013	USA	NO_2_	Prospective, year-long study	1,342	Children	Asthma severity, wheezing, night symptoms due to asthma, medication use due to asthma
Kloog (12)	2013	USA	PM_2.5_	Short-term exposure	46,8,570	All	PM-related mortality
Raaschou-Nielsen (46)	2013	Europe	PM_2.5_, PM_10_	Cohort study	312,944	All	Lung cancer, adenocarcinoma
Cortez-Lugo (1)	2015	Mexico	PM_2.5_	Questionnaire	29	Adults	COPD cough, COPD phlegm
Fan (8)	2016	China	PM_2.5_	Meta-analysis	16	Children, adults, all	Asthma ED
Ghozikali (47)	2016	Iran	NO_2_, O_3_, SO_2_	Unknown	Unknown	All	COPD hospitalizations, COPD hospitalization, COPD HA
Greenberg (48)	2016	Israel	SO_2_	Exposure assessment	137,040	Children	Asthma severity
Hasunuma (49)	2016	Japan	NO_2_	Nested case–control study	853 (case),3,409 (control)	Children	Persistence of asthmatic symptoms
Mirabelli (50)	2016	USA	PM_2.5_	Call-back survey	50,356	Adults	Any asthma symptoms
Pollitt (51)	2016	Canada	Aluminum, iron, magnesium, sulfur, nickel, vanadium, Chromium, arsenic, manganese, barium, copper, antimony, zinc	Questionnaire	217	Children	Airway inflammation due to asthma
Li (15)	2016a	China	NO_2_, SO_2_	Meta-analysis	59	All	COPD exacerbations
Li (52)	2016b	China	SO_2_	Unknown	10,095	Adults	COPD mortality
Day (53)	2017	China	O_3_	Longitudinal study	89	Adults	Pulmonary inflammation
Khaniabadi (54)	2017	Iran	O_3_	Unknown	Unknown	All	Cardiopulmonary mortality, COPD hospitalization
Lamichhane (32)	2018	India	PM_2.5_, PM_10_, NO_2_	Unknown	1,264	Adults	Reduced lung function due to COPD
Magzamen (55)	2018	USA	PM_10_, NO_2_	Unknown	35	Adults	Inhaler use due to COPD
Mao (34)	2018	China	Copper, iron	Case–control study	33	All	Asthma susceptibility
Hansel (56)	2019	USA	PM_2.5_	Longitudinal study	484	Children	Uncontrolled asthma
Huang (57)	2019	Taiwan	PM_2.5_	Exposure assessment	3,941	Adults	Susceptibility to COPD
Wu (7)	2019	USA	Lead	Cross-sectional, population-based study	5,866	Children	Risk for active asthma, wheezing
Mercan (58)	2020	Turkey	SO_2_	Unknown	710	Adults	Asthma hospitalization, COPD hospitalization
Pepper (59)	2020	USA	O_3_	Randomized controlled trial	287	Children, adults	Asthma rescue inhaler use
Yu (14)	2020	Australia	PM_2.5_	Meta-analysis	242,320	all	Respiratory mortality
Shin (60)	2021	Canada	PM_2.5_, PM_10_, NO_2_	Population-based study	558,738	Adults	COPD
Yan (61)	2022	China	PM_2.5_	Retrospective	39,054	All	Chronic bronchitis, asthma, COPD, chronic respiratory disease
Mebrahtu (62)	2023	UK	PM_2.5_, PM_10_, NO_2_	Retrospective	124,808	All	Respiratory illnesses
Aron (63)	2023	USA	PM_2.5_	Time stratified case-crossover	19,243	Adults	COPD
Kangas (64)	2023	Belgium	PM_2.5_	Census	437,340	Adults	Asthma
Marchetti (65)	2023	Italy	PM_2.5_, PM_10,_ NO_2_	Multi case–control	16,173	Adults	Rhinitis, COPD
Wang (66)	2023	Taiwan	Arsenic	Cross-sectional	1,563	Adults	Lung fibrotic, bronchiectasis
Yang (67)	2024	China	O_3_	Retrospective	4,574	all	COPD
Stowell (68)	2024	USA	O_3_	One state case-crossover	111,71,779	Children	Allergy, asthma, respiratory disorders, respiratory infections
Yu (69)	2024	China	Cu, Zn, Se	Survey	2,807	Adults	COPD, emphysema, tracheitis
Kwon (70)	2024	South Korea	NO_2_	Survey	22,387	Adults	COPD
Zhang (71)	2024	China	O_3_	Prospective cohort	10,973	Adults	Asthma
Zheng (72)	2024	China	PM_2.5_, PM_10_	Multistage probability based	7,371	All	Asthma, wheezing, dyspnea
Xing (73)	2024	China	O_3_	Population-based study	6,537	all	COPD

COPD, chronic obstructive pulmonary disease; ED, emergency department; HA, hospital admission; PM, particulate matter; PIAMA, prevention and incidence of asthma and mite allergy.

### 3.2 Effects of PM_2.5_ and PM_10_ exposure increase

**Figure 2:**
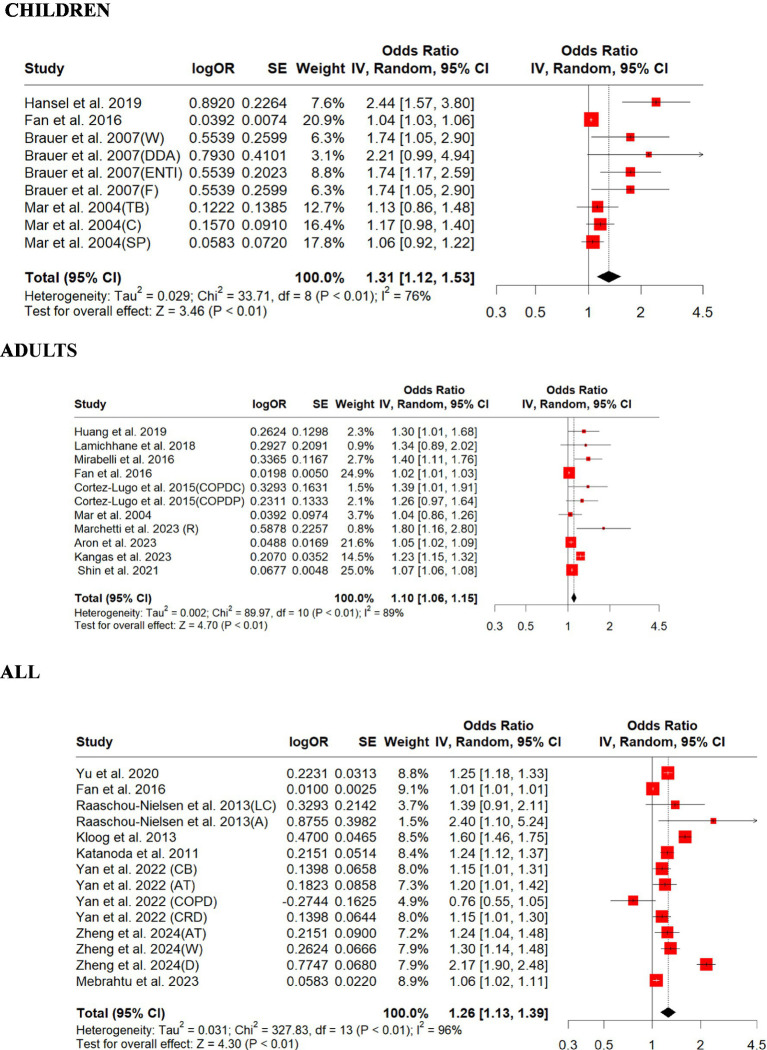
Forest plot of the estimated effects of PM_2.5_ on respiratory disease. A, adenocarcinoma; AT, asthma; C, coughing; CB, chronic bronchitis; COPDC, COPD cough; COPDP, COPD phlegm; CRD, chronic respiratory disease; D, dyspnea; DDA, doctor-diagnosed asthma; ENTI, ear/nose/throat infections; F, flu; LC, lung cancer; TB, trouble breathing; SP, sputum production; R, rhinitis; W, wheezing.

We included 12 PM_2.5_ and 6 PM_10_ studies in this review and summarized their findings here. Our standardized effect estimates showed that the associations (ORs) of PM_2.5_ with respiratory outcomes ranged from 0.76 to 2.44 per 10 μg/m^3^ increase in exposure (Figure 2). Children had significant overall OR (1.31, 95% CI: 1.12–1.53, *p* = 0.0005); adults also had significant overall OR (1.10, 95% CI: 1.06–1.15, *p* = 0.0001; Figure 2). For those studies that did not differentiate children from adults, the overall OR (1.26, 95% CI: 1.13–1.39, *p* = 0.0001) was significant (Figure 2). There was strong evidence of heterogeneity among these studies (children, *Q* = heterogeneity *χ*^2^ = 33.71, *I*^2^ = 76%, *p* = 0.0001; adults, *Q* = heterogeneity *χ*^2^ = 89.97, *I*^2^ = 89%, *p* = 0.0001; all, *Q* = heterogeneity *χ*^2^ = 327.83, *I*^2^ = 96%, *p* = 0.0001; Figure 2). Egger’s linear regression did not identify publication bias among the studies for adults (Egger’s: *p* = 0.087), but for children (Egger’s: *p* = 0.002) and for all (Egger’s: *p* = 0.003; Supplementary Figure S1). The children’s original model estimate is 0.2714, and the overall sensitivity analysis model estimate is slightly lower at 0.2497 (Supplementary Table S2). Both estimates indicate a positive association of PM_2.5_ with respiratory outcomes in children. The sensitivity analysis shows that the meta-analysis results are robust, as the effect size estimates remain statistically significant regardless of which study is excluded. The original and overall models show consistent results, reinforcing the conclusion that PM_2.5_ exposure is associated with respiratory disease in children (Supplementary Table S1). The effect size estimate is 0.0960 for both the original and overall models, indicating a consistent positive association of PM_2.5_ with respiratory outcomes in adults (Supplementary Table S4). The original and overall models show consistent results, reinforcing the conclusion that PM_2.5_ exposure is associated with respiratory disease in adults (Supplementary Table S3). The effect size estimate is 0.2283 for the original model and slightly lower at 0.2186 for the overall sensitivity analysis model, indicating a consistent positive association of PM_2.5_ with respiratory disease across all age groups (Supplementary Table S6). The sensitivity analysis shows that the meta-analysis results are robust, as the effect size estimates remain statistically significant regardless of which study is excluded. The original and overall models show consistent results, reinforcing the conclusion that PM_2.5_ exposure is associated with respiratory disease across all age groups (Supplementary Table S5).

**Figure 3:**
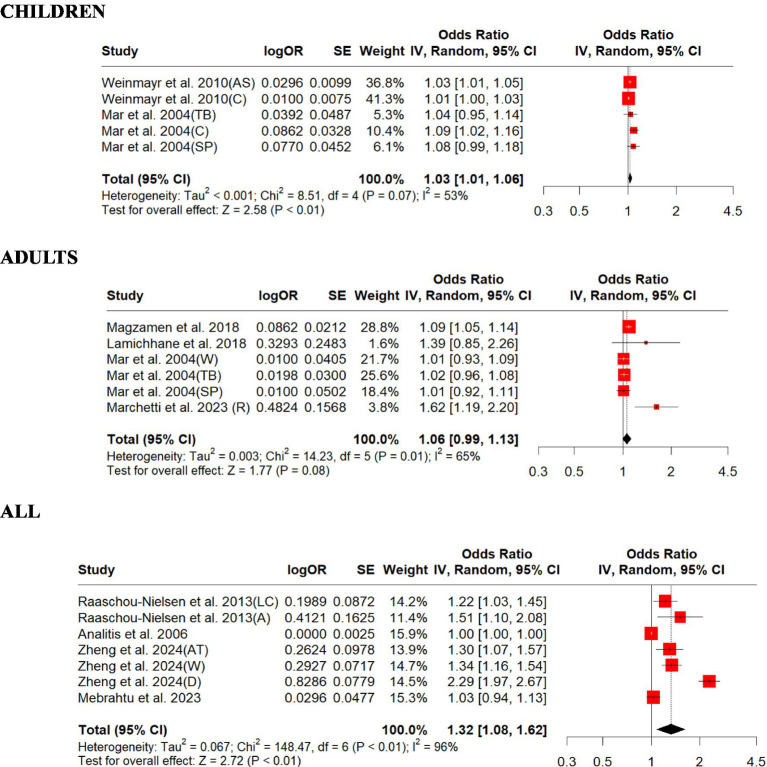
Forest plot of the estimated effects of PM_10_ on respiratory disease. A, adenocarcinoma; AS, asthma symptoms, AT, asthma; C, coughing, D, dyspnea; LC, lung cancer; R, rhinitis; SP, sputum production; TB, trouble breathing; W, wheezing.

For PM_10_, the association (ORs) ranged from 1.00 to 2.29 per 10 μg m^−3^ increase in exposure. Children were found to have a relatively lower overall OR (1.03, 95% CI: 1.01–1.06, *p* = 0.01) than adults (1.06, 95% CI: 0.99–1.13, *p* = 0.07; Figure 3). For those studies that did not differentiate children from adults, the overall OR (1.32, 95% CI: 1.08–1.62, *p* = 0.006) was slightly higher than those for children and adults (Figure 3). There was strong evidence of homogeneity for children (children, *Q* = heterogeneity *χ*^2^ = 8.50, *I*^2^ = 53%, *p* = 0.07), but heterogeneity was found for those studies that did not differentiate all from adults (adults, *Q* = Heterogeneity *χ*^2^ = 14.22, *I*^2^ = 65%, *p* = 0.01; all, *Q* = heterogeneity *χ*^2^ = 148.47, *I*^2^ = 96%, *p* = 0.0001; Figure 3). Egger’s linear regression did identify publication bias among the studies for all (Egger’s: *p* = 0.04), and children (Egger’s: *p* = 0.11) but did not identify publication bias for adults (Egger’s: *p* = 0.46; Supplementary Figure S2). The effect size estimate is 0.0308 for both the original and overall models, indicating a consistent positive effect of PM_10_ on respiratory outcomes in children (Supplementary Table S8). The sensitivity analysis shows that the meta-analysis results are robust, as the effect size estimates remain statistically significant regardless of which study is excluded. The original and overall models show consistent results, reinforcing the conclusion that PM_10_ exposure is associated with respiratory outcomes in children (Supplementary Table S7). The effect size estimate is 0.0576 for both the original and overall models, indicating a small positive effect of PM_10_ on respiratory outcomes in adults (Supplementary Table S10). The sensitivity analysis shows that the meta-analysis results are robust, as the effect size estimates remain consistent regardless of which study is excluded. However, the original and overall models show that the effect of PM_10_ on respiratory outcomes in adults is not statistically significant, with confidence intervals that include zero and a *p*-value slightly above 0.05 (Supplementary Table S9). This suggests that further research might be needed to establish a clearer relationship. The effect size estimate is 0.2798 for both the original and overall models, indicating a moderate positive effect of PM_10_ on respiratory disease across all age groups (Supplementary Table S12). The sensitivity analysis shows that the meta-analysis results are robust, as the effect size estimates remain consistent regardless of which study is excluded. The original and overall models show a statistically significant positive effect of PM_10_ on respiratory disease across all age groups, with moderate heterogeneity. This suggests a meaningful relationship between PM_10_ exposure and respiratory disease, warranting further attention and research (Supplementary Table S11).

### 3.3 Impacts from nitrogen dioxide exposure increase

**Figure 4:**
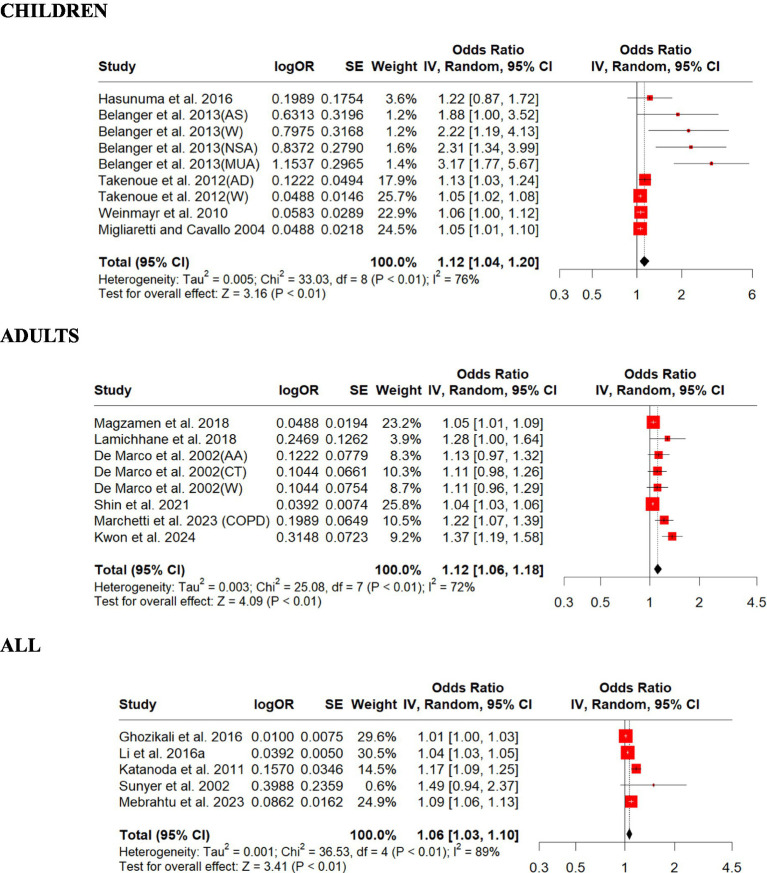
Forest plot of the estimated effects of NO_2_ on respiratory disease. AA, asthma attack; AD, asthma development; AS, asthma severity; CT, chest tightness; MUA. medication use due to asthma; NSA, night symptoms due to asthma; W, wheezing.

We included 12 studies on NO_2_ in this review and summarized their findings here. Our standardized estimates showed that NO_2_ associations (ORs) ranged from 1.01 to 3.17 per 10 ppb increase in exposure. Children were found to have a relatively lower overall OR (1.12, 95% CI: 1.04–1.20, *p* = 0.001) than adults (1.12, 95% CI: 1.06–1.18, *p* = 0.0001; Figure 4). The overall all age group OR (1.06, 95% CI: 1.03–1.10, *p* = 0.0016) was slightly higher than that of children and adults (Figure 4). There was strong evidence of heterogeneity among these studies (children, *Q* = heterogeneity *χ*^2^ = 33.03, *I*^2^ = 76%, *p* = 0.0001; adults, *Q* = heterogeneity *χ*^2^ = 25.08, *I*^2^ = 72%, *p* = 0.0001; all, *Q* = heterogeneity *χ*^2^ = 36.53, *I*^2^ = 89%, *p* = 0.0001; Figure 4). Egger’s linear regression did identify publication bias among the studies for adults (Egger’s: *p* = 0.008) and children (Egger’s: *p* = 0.0005) but did not identify publication bias for all (Egger’s: *p* = 0.24; Supplementary Figure S3). The effect size estimate is 0.1137 for the original model and slightly lower at 0.0911 for the overall sensitivity analysis model, indicating a positive effect of NO_2_ on respiratory disease in children (Supplementary Table S14). The sensitivity analysis shows that the meta-analysis results are robust, as the effect size estimates remain statistically significant regardless of which study is excluded. The original and overall models show consistent results, reinforcing the conclusion that NO_2_ exposure is associated with respiratory disease in children (Supplementary Table S13). The effect size estimate is 0.1110 for both the original and overall models, indicating a positive effect of NO_2_ on respiratory disease in adults (Supplementary Table S16). The sensitivity analysis shows that the meta-analysis results are robust, as the effect size estimates remain consistent regardless of which study is excluded. The original and overall models show a statistically significant positive effect of NO_2_ on respiratory disease in adults, with moderate heterogeneity. This suggests a meaningful relationship between NO_2_ exposure and respiratory disease, warranting further attention and research (Supplementary Table S15). The effect size estimate is 0.0614 for both the original and overall models, indicating a positive effect of NO_2_ on respiratory disease across all age groups (Supplementary Table S18). The sensitivity analysis shows that the meta-analysis results are robust, as the effect size estimates remain consistent regardless of which study is excluded. The original and overall models show a statistically significant positive effect of NO_2_ on respiratory disease across all age groups, with moderate heterogeneity. This suggests a meaningful relationship between NO_2_ exposure and respiratory disease, warranting further attention and research (Supplementary Table S17).

### 3.4 Impacts of ozone exposure increase

**Figure 5:**
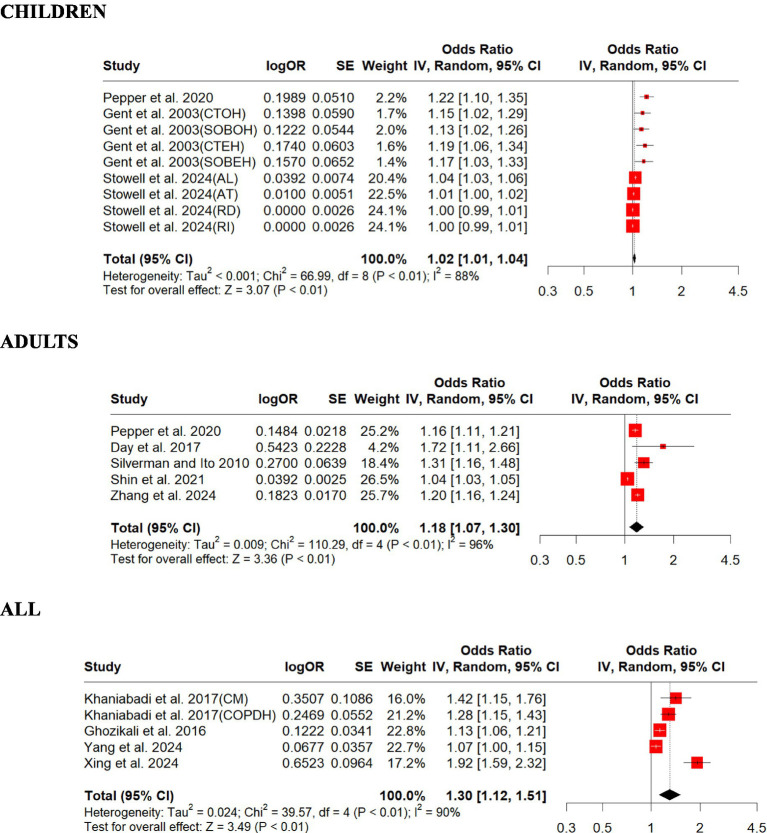
Forest plot of the estimated effects of O_3_ on respiratory disease. AL, allergy; AT, asthma; CM, cardiopulmonary mortality; COPDH, COPD hospitalization; CTEH, chest tightness (8 h); CTOH, chest tightness (1 h); RD, respiratory disorders; RI, respiratory infections; SOBEH, shortness of breath (8 h); SOBOH, shortness of breath (1 h).

We included 6 studies on O_3_ in this review and summarized their findings here. Our standardized estimates showed that O_3_ associations (ORs) ranged from 1.00 to 1.92 per a 30-ppb increase in exposure. Children were found to have lower overall OR (1.02, 95% CI: 1.01–1.04, *p* = 0.001) compared to adults (1.18, 95% CI: 1.07–1.30, *p* = 0.001; Figure 5). For those studies that did not differentiate children from adults, all age overall OR (1.30, 95% CI: 1.12–1.51, *p* = 0.001) was slightly higher than those for children and adults (Figure 5). There was strong evidence of homogeneity among these studies (adults, *Q* = heterogeneity *χ*^2^ = 110.29, *I*^2^ = 96%, *p* = 0.09; all, *Q* = heterogeneity *χ*^2^ = 39.57, *I*^2^ = 90%, *p* = 0.05), but heterogeneity was found for children (children, *Q* = heterogeneity *χ*^2^ = 66.99, *I*^2^ = 88%, *p* = 0.0001; Figure 5). Egger’s linear regression did identify publication bias among the studies for all (Egger’s: *p* = 0.04) but did not identify publication bias for children (Egger’s: *p* = 0.5) and adults (Egger’s: *p* = 0.05; Supplementary Figure S4). The effect size estimate is 0.0244 for both the original and overall models, indicating a small positive effect of O_3_ on respiratory disease in children (Supplementary Table S20). The sensitivity analysis shows that the meta-analysis results are robust, as the effect size estimates remain consistent regardless of which study is excluded. The original and overall models show a statistically significant positive effect of O_3_ on respiratory disease in children, with moderate heterogeneity. This suggests a meaningful relationship between O_3_ exposure and respiratory disease, warranting further attention and research (Supplementary Table S19). The effect size estimate is 0.1672 for both the original and overall models, indicating a positive effect of O_3_ on respiratory disease in adults (Supplementary Table S22). The sensitivity analysis shows that the meta-analysis results are robust, as the effect size estimates remain consistent regardless of which study is excluded. The original and overall models show a statistically significant positive effect of O_3_ on respiratory disease in adults, with high heterogeneity. This suggests a meaningful relationship between O_3_ exposure and respiratory disease, warranting further attention and research (Supplementary Table S21). The effect size estimate is 0.2643 for both the original and overall models, indicating a positive effect of O_3_ on respiratory disease across all age groups (Supplementary Table S24). The sensitivity analysis shows that the meta-analysis results are robust, as the effect size estimates remain consistent regardless of which study is excluded. The original and overall models show a statistically significant positive effect of O_3_ on respiratory disease across all age groups, with high heterogeneity. This suggests a meaningful relationship between O_3_ exposure and respiratory disease, warranting further attention and research (Supplementary Table S23).

### 3.5 Impacts from sulfur dioxide exposure increase

**Figure 6:**
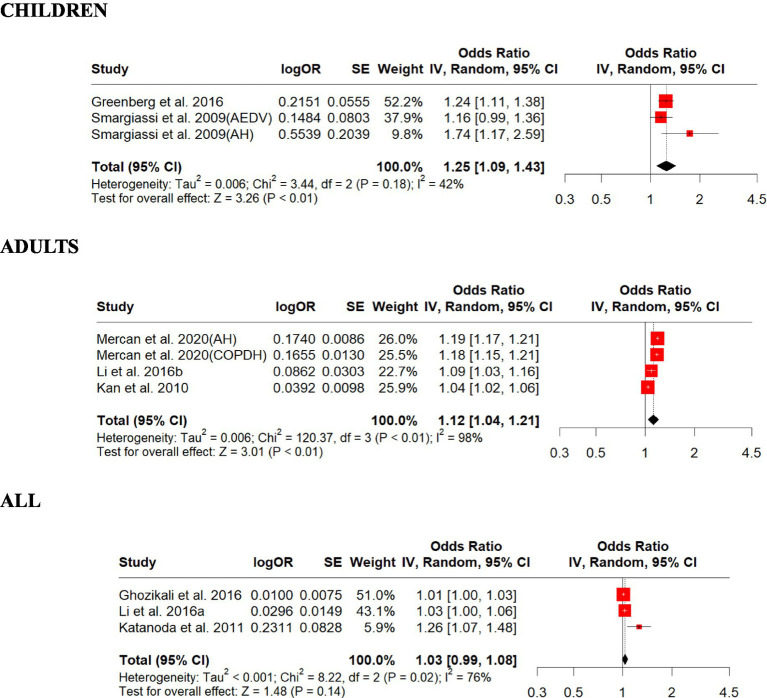
Forest plot of the estimated effects of SO_2_ on respiratory disease. AH, asthma hospitalization; AEDV, asthma emergency department visits; COPDH, COPD hospitalization.

We included eight studies on SO_2_ in this review and summarized their findings here. Our standardized estimates showed that SO_2_ associations (ORs) ranged from 1.01 to 1.74 per 10-ppb increase in exposure. Children were found to have the highest overall OR (1.25, 95% CI: 1.09–1.43, *p* = 0.001) than adults (1.12, 95% CI: 1.04–1.21, *p* = 0.002; Figure 6). For those studies that did not differentiate children from adults, the overall OR (1.03, 95% CI: 0.99–1.08, *p* = 0.13) was slightly lower than those for children and adults (Figure 6). There was strong evidence of homogeneity among these studies (children, *Q* = heterogeneity *χ*^2^ = 3.44, *I*^2^ = 42%, *p* = 0.18), but heterogeneity was found for adults and all (adults, Q = Heterogeneity *χ*^2^ = 120.37, *I*^2^ = 98%, *p* = 0.0001; all, *Q* = heterogeneity *χ*^2^ = 8.22, *I*^2^ = 76%, *p* = 0 0.01; Figure 6). Egger’s linear regression did identify publication bias among the studies for all (Egger’s: *p* = 0.023) but did not identify children (Egger’s: *p* = 0.47) and adults (Egger’s: *p* = 0.88; Supplementary Figure S5). The effect size estimate is 0.2232 for both the original and overall models, indicating a positive effect of SO_2_ on respiratory disease in children (Supplementary Table S26). The sensitivity analysis shows that the meta-analysis results are robust, as the effect size estimates remain consistent regardless of which study is excluded. The original and overall models show a statistically significant positive effect of SO_2_ on respiratory disease in children, with moderate heterogeneity. This suggests a meaningful relationship between SO_2_ exposure and respiratory disease, warranting further attention and research (Supplementary Table S25). The effect size estimate is 0.1171 for both the original and overall models, indicating a positive effect of SO_2_ on respiratory disease in adults (Supplementary Table S28). The sensitivity analysis shows that the meta-analysis results are robust, as the effect size estimates remain consistent regardless of which study is excluded. The original and overall models show a statistically significant positive effect of SO_2_ on respiratory disease in adults, with high heterogeneity. This suggests a meaningful relationship between SO_2_ exposure and respiratory disease, warranting further attention and research (Supplementary Table S27). The effect size estimate is 0.0315 for both the original and overall models, indicating a small positive effect of SO_2_ on respiratory disease across all age groups (Supplementary Table S30). The sensitivity analysis shows that the meta-analysis results are robust, as the effect size estimates remain consistent regardless of which study is excluded. However, the original and overall models show that the effect of SO_2_ on respiratory disease across all age groups is not statistically significant, with confidence intervals that include zero and a *p*-value above 0.05. This suggests that further research might be needed to establish a more transparent relationship (Supplementary Table S29).

### 3.6 Impacts from trace metals from on-road vehicle emissions

We included three studies on trace metals in this review and summarized their findings here.

**Figure 7:**
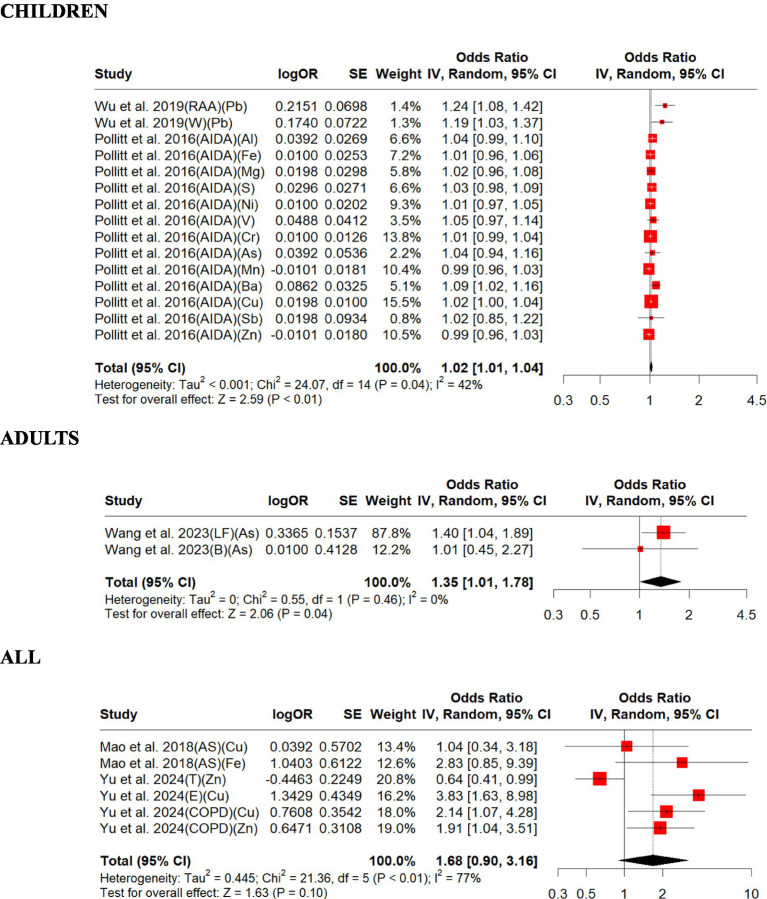
Forest plot of the estimated effects of trace metal on respiratory disease. AIDA, airway inflammation due to asthma; Al, aluminum; As, arsenic; AS, asthma susceptibility; B, bronchiectasis; Ba, barium; Cr, chromium; Cu, copper; E, emphysema; Fe, iron; LF, lung fibrotic; Pb, lead; Mg, magnesium; Mn, manganese; Ni, nickel; RAA, risk for active asthma; S, sulfur; Sb, antimony; T, tracheitis; W, wheezing; Zn, zinc.

Overall, the studies included in this review support that increased exposures to trace metals are associated with exacerbations in respiratory disease outcomes (ORs from 1.01 to 3.83) except for two cases (ORs = 0.99). The exacerbations in the respiratory disease outcomes in children and all subjects were significantly associated with increased exposure to trace metals. Children were found to have a lower overall OR (1.02, 95% CI: 1.01–1.04, *p* = 0.009) than all adults (1.35, 95% CI: 1.01–1.78, *p* = 0.039) and all (1.68, 95% CI: 0.90–3.16, *p* = 0.104; Figure 7). There was strong evidence of homogeneity for adults (adults, *Q* = heterogeneity *χ*^2^ = 0.55, I^2^ = 0%, *p* = 0.45), but heterogeneity found for children and all (children, *Q* = heterogeneity *χ*^2^ = 24.07, *I*^2^ = 42%, *p* = 0.04; all, *Q* = Heterogeneity *χ*^2^ = 21.36, *I*^2^ = 77%, *p* = 0.0007; Figure 7). Egger’s linear regression did not identify publication bias for all (Egger’s: *p* = 0.15) but for children (Egger’s: *p* = 0.02; Supplementary Figure S6). The effect size estimate is 0.0220 for both the original and overall models, indicating a small positive effect of trace metals on respiratory disease in children (Supplementary Table S32). The sensitivity analysis shows that the meta-analysis results are robust, as the effect size estimates remain consistent regardless of which study is excluded. The original and overall models show a statistically significant positive effect of trace metals on respiratory disease in children, with moderate heterogeneity. This suggests a meaningful relationship between trace metal exposure and respiratory disease, warranting further attention and research (Supplementary Table S31). The effect size estimate is 0.2967 for the original model and slightly higher at 0.3365 for the overall sensitivity analysis model, indicating a positive effect of trace metals on respiratory disease in adults (Supplementary Table S34). The sensitivity analysis shows that the meta-analysis results are robust, as the effect size estimates remain statistically significant regardless of which study is excluded. The original and overall models show a statistically significant positive effect of trace metals on respiratory disease in adults, with no heterogeneity. This suggests a meaningful relationship between trace metal exposure and respiratory disease, warranting further attention and research (Supplementary Table S33). The effect size estimate is 0.5215 for the original model and much lower at 0.0794 for the overall sensitivity analysis model. This indicates that the initial model suggested a moderate positive effect of trace metals on respiratory disease across all age groups, but the sensitivity analysis drastically reduces this effect size (Supplementary Table S36). The sensitivity analysis shows that the meta-analysis results are not robust, as the effect size estimates vary significantly depending on which study is excluded. The original and overall models both indicate that the effect of trace metals on respiratory disease across all age groups is not statistically significant, with confidence intervals that include zero and high heterogeneity. This suggests that there is no clear relationship between trace metal exposure and respiratory disease based on the available data, and further research with more consistent findings is needed (Supplementary Table S35).

## 4 Discussion

In this meta-analysis, we assessed the impact of exposures to criteria pollutants (NO_2_, PM_2.5_, PM_10_, O_3_, and SO_2_) and more than 10 trace metals that can be considered toxic air contaminants on respiratory disease outcomes, including airway inflammation, coughing, wheezing, exacerbations, ED visits, hospitalizations, and mortality. We converted all the study outcomes in RRs and percentage increase to ORs and standardized the impact of exposure increase interval to make the studies comparable. After standardizing the results from different studies, we pooled the effect estimates to report the overall size impact of PM_2.5_ (Figure 2) and PM_10_ (Figure 3) on respiratory disease outcomes.

A previous systematic review on the association of major air pollutants with the risk of COPD concludes that even short-term exposures to air pollutants significantly increase the risk of COPD acute exacerbations (15). Furthermore, a recent study found that NO_2_ or PM_2.5_ concentration in nine southern Californian communities had been lower than observed in the 1990s and early 2000s; there would have been a corresponding reduction in childhood asthma incidence (31). A meta-analysis study found clear evidence of an association of PM_10_ exposure with asthma symptoms and, to a lesser extent, with cough and peak expiratory flow (PEF) (3). Another previous study used linear and logistic regression analyses to investigate the associations between chronic exposure to PM_10_, PM_2.5_, and NO_2_ levels and lung function. The study provided evidence that exposure to ambient air pollution adversely affects adult lung function (32). A previous study showed every 5 ppb increase in NO_2_ exposure above a threshold of 6 ppb was associated with an exposure-dependent rise in the risk of higher asthma severity score, wheezing, night symptoms, and rescue medication use (4). A previous review of the health effects caused by environmental NO_2_ reported that short-term (24-h) exposure to NO_2_, even for mean values <50 μg/m^3^, increased both hospital admission and mortality (13). It also reported that long-term exposure to the NO_2_ level below the WHO recommended air quality annual mean guideline of 40 μg/m^3^ was associated with adverse health effects such as respiratory symptoms and diseases, hospitalizations, and mortality (13). A previous study shows that even living in an area with air pollution concentrations below the current US Environmental Protection Agency-National Ambient Air Quality Standard (US EPA-NAAQS), COPD patients suffered an increased risk of exacerbations following short-term exposures to increased concentrations of SO_2_ (33). Compared with other criteria pollutants, the impact of trace metals on human respiratory health has not been studied much. Based on a previous study, trace elements may be involved in the pathogenesis of asthma (34). Metals in tailpipe and non-tailpipe emissions, such as brake and tire wear, include copper, zinc, antimony, barium, lead, and sulfur (35). A previous study showed that daily exposures to ambient PM_2.5_ containing trace metals (barium and vanadium) are associated with airway inflammation in asthmatic children. Results from another study suggest that high ambient air PM_2.5_ containing zinc is associated with increased emergency department visits and hospitalizations for asthma among children living in an urban area (9).

We identified consistently significant associations between all the criteria pollutants and trace metals with a broad range of respiratory disease outcomes. After pooling all the effect estimates, we further identified that the associations were slightly higher for children for exposures to SO_2_ (1.25 vs. 1.12) and PM_2.5_ (1.31 vs. 1.10) than for adults but with similar associations for children and adults for exposures to NO_2_ (1.12 vs. 1.12) while the effects were slightly higher for adults for exposures to PM_10_ (1.03 vs. 1.06) and O_3_ (1.02 vs. 1.18) than for children.

Outdoor NO_2_ concentration is often considered a good marker of traffic-related air pollution (TRAP), and concentrations decline rapidly with increasing distance from highways and major roadways. PM_2.5_ has smaller spatial gradients than NO_2_, and regional sources contribute to PM_2.5_ concentrations. However, traffic remains a major source of PM_2.5_, especially in high-income countries. SO_2_ air pollution can be generated from traffic, especially diesel vehicles, and industrial point sources such as coal-fired power plants, rapidly declining with increasing distances from these point sources.

The relatively higher impact of NO_2_, PM_2.5_, and SO_2_ on respiratory disease outcomes for children may be due to both biological and environmental factors. Biologically, children’s lungs are still developing, making them more susceptible to damage from pollutants (36). Environmentally, children are more likely to spend time outdoors and engage in physical activities in areas with high pollutant concentrations, such as near schools and playgrounds in urban settings. This increased exposure during critical growth periods can have both short-term and long-term adverse effects on their respiratory health. Policymakers and stakeholders should adopt strategies to mitigate children’s exposure to air pollution, especially in vulnerable communities. This can include implementing stricter air quality standards around schools and playgrounds, promoting urban planning policies that minimize children’s exposure to high-traffic areas, and encouraging the development of green spaces to act as buffers against pollution. Addressing these factors can help protect children’s health and reduce the burden of respiratory diseases linked to air pollution.

An OR greater than 1.0 indicates an increased risk, while an OR less than 1.0 suggests a protective effect. When ORs are close to 1.0 or when confidence intervals (CIs) include 1.0, it implies a weak or non-significant association. However, even small effect sizes can be meaningful in a public health context due to widespread exposure and the cumulative impact of multiple pollutants. Small increases in risk can lead to significant health impacts across large populations, particularly for vulnerable groups such as children and the older adult. The impact of O_3_ on respiratory disease outcomes was found to be relatively higher for adults. This may be due to the chemical reactions between O_3_ and traffic emissions of nitrogen oxides, which lead to higher NO_2_ concentrations near busy roadways and higher O_3_ levels farther from traffic. We suspect more adults might have moved to communities with less traffic-related air pollution, thus experiencing greater O_3_ exposure. Policymakers need to consider these findings to make informed decisions. Non-significant findings should be interpreted cautiously and not used in isolation to justify policy changes. Continuous monitoring and research are essential to understand health impacts better and refine policy interventions. Therefore, the practical implications of our findings are significant for shaping effective public health policies and mitigating respiratory health risks. Therefore, policymakers might consider precautionary measures to limit exposure, especially if the pollutant has other adverse health effects or the population is particularly vulnerable (e.g., children and older adult).

To address the critical need for urban planning and transportation policies that link urban air pollution and respiratory health, as highlighted in this meta-analysis, urban planning must integrate green infrastructure like parks and green roofs, which can absorb pollutants and reduce urban heat island effects. Transportation policies should prioritize the development of public transit options and non-motorized transport to decrease reliance on private vehicles, thereby reducing non-exhaust emissions from road traffic, such as brake and tire wear. Specific policies could include implementing low-emission zones, encouraging the use of electric or hybrid vehicles, and investing in regular road maintenance to reduce road dust resuspension. Regarding climate action, integrated approaches can be designed to reduce emissions from both transportation and industrial sources by promoting renewable energy, electrifying transport systems, and implementing stricter emissions standards. Expanding green urban spaces can serve dual purposes of carbon sequestration and pollution mitigation. This meta-analysis provides critical evidence to inform the development of such integrated policies by quantifying the health impacts of specific pollutants, emphasizing the urgency of reducing emissions to improve public health and climate resilience simultaneously.

### 4.1 Limitations and the future directions

To maintain an adequate sample size, we combined all studies for each criterion pollutant, regardless of the type of outcome measured, and compared the results across different outcomes. This study assessed the effects of airborne particulate matter (PM_2.5_ and PM_10_), NO_2_, O_3_, SO_2_, and trace metals. While this comprehensive approach provides a broad understanding, it introduces considerable heterogeneity, as indicated by the Higgins *I*^2^ values. In addition to assessing methodological quality, we evaluated the risk of bias for each study using the Newcastle–Ottawa scale (NOS). The study locations of the 50 included studies were highly heterogeneous, spanning both developed and developing countries across several continents, limiting our findings’ generalizability. It is also worth noting that the impact of trace metals on human respiratory health is understudied, with only five studies included in the meta-analysis. Furthermore, the role of trace metals on adults could barely be assessed due to the limited number of studies focusing on this demographic; most reports concentrated on children or combined age groups.

Although we had five studies in our trace metals analysis, the limited number of studies still requires a cautious interpretation of the results. This small sample size highlights the need for more research to better understand the impact of trace metals on respiratory health. The future studies should focus on expanding the evidence base to ensure more reliable and generalizable findings. More comprehensive research is essential to assess the risks and inform effective public health policies accurately. Another limitation is the lack of information on confounding factors, which could affect the robustness of the results.

The future research should aim to conduct more studies that include specific chemical components of particulate matter, monitor the same individuals over time, and adjust for confounding variables. This approach would enhance our understanding of the relationship between air pollution and respiratory outcomes and provide more accurate data to inform policymakers. Additionally, analyzing long-term and short-term exposures separately could provide more precise risk assessments and clearer insights into specific health outcomes associated with each type of exposure.

## 5 Conclusion

The findings from this meta-analysis support a positive, robust association between criteria air pollutants, trace metals, and respiratory disease, indicating significant impacts across all age groups, including children and adults. Our study highlights the importance of considering both traditional criteria pollutants and non-exhaust trace metals, which showed significant effects on respiratory health despite being studied in a smaller number of research efforts. This underscores the need to include these often-overlooked pollutants in the future research studies to comprehensively understand the environmental determinants of respiratory disease.

We recommend that the future clinical and epidemiological studies elucidate the potential mechanisms underlying these positive associations. This involves exploring the biological pathways through which air pollutants and trace metals contribute to respiratory conditions. By understanding these mechanisms, more targeted and effective interventions can be developed.

Given our findings, it is crucial to implement rigorous environmental health policies to maintain low levels of air pollution. Such policies are vital to reducing the incidence and severity of respiratory diseases and lowering morbidity and mortality associated with air pollution exposure. Governments should prioritize air quality regulations and ensure strict enforcement to protect public health. A collaborative approach is essential to address the multifaceted nature of air pollution. This involves cooperation between government entities, the automobile industry, the energy sector, and transportation companies. Promoting cleaner fuels, such as natural gas and electricity, can significantly reduce emissions from transportation, one of the significant sources of air pollutants. Additionally, investment in renewable energy sources and sustainable urban infrastructure development are critical components of a comprehensive strategy to combat air pollution.

Further studies are warranted from countries with higher ambient air pollution levels, often facing the most severe health impacts. These studies should provide more comprehensive data on the association between air pollution and respiratory disease, considering different geographic locations’ unique environmental and socioeconomic contexts.

In conclusion, the robust associations identified in this meta-analysis emphasize the urgent need for concerted efforts to mitigate air pollution and its adverse effects on respiratory health. By advancing research, strengthening policies, and fostering cross-sector collaboration, we can achieve substantial progress in reducing air pollution levels and safeguarding public health for the future generations.
