# Supplementary material for: Impacts from air pollution on respiratory disease outcomes: a meta-analysis
Source: Front Public Health. 2024 Oct 9;12:1417450. doi: 10.3389/fpubh.2024.1417450 (PMC11497638; doi:10.3389/fpubh.2024.1417450)
Supplement: Supplementary file 1 [file Data_Sheet_1.docx]

Supplementary Materials

**TABLE S 1 – 36** Sensitivity analysis

**TABLE S 37** Newcastle-Ottawa risk of bias scoring

**FIGURE S 1-6** Funnel plot of the Odds ratio of respiratory disease

**TABLE S 1**  Sensitivity analysis of respiratory disease of PM 2.5 for children

| Excluded Study | Effect Size Estimate | Standard Error | Z-value | P-value |
| --- | --- | --- | --- | --- |
| "Hansel et al. 2019" | 0.18 | 0.06 | 2.83 | 0.004 |
| "Fan et al. 2016" | 0.36 | 0.10 | 3.50 | 0.0004 |
| "Brauer et al. 2007(W)" | 0.24 | 0.07 | 3.13 | 0.001 |
| "Brauer et al. 2007(DDA)" | 0.24 | 0.07 | 3.22 | 0.001 |
| "Brauer et al. 2007(ENTI)" | 0.23 | 0.07 | 3.01 | 0.002 |
| "Brauer et al. 2007(F)" | 0.24 | 0.07 | 3.13 | 0.001 |
| "Mar et al. 2004(TB)" | 0.30 | 0.08 | 3.44 | 0.0005 |
| "Mar et al. 2004(C)" | 0.31 | 0.09 | 3.33 | 0.0008 |
| "Mar et al. 2004(SP)" | 0.35 | 0.10 | 3.38 | 0.0007 |

**TABLE S 2** PM 2.5 sensitivity analysis model summaries for children

| Model | Effect Size Estimate | Standard Error | Z-Value | P-Value | ci.lb | ci.ub | tau^2 | I^2 |
| --- | --- | --- | --- | --- | --- | --- | --- | --- |
| Original | 0.2714 | 0.0784 | 3.4604 | 0.0005 | 0.1177 | 0.4250 | 0.0293 | 76.27% |
| Overall | 0.2497 | 0.0775 | 3.2240 | 0.0013 | 0.0979 | 0.4015 | 0.0270 | 76.95% |
|  |  |  |  |  |  |  |  |  |

**TABLE S 3** Sensitivity analysis of respiratory disease of PM 2.5 for adults

| Excluded Study | Effect Size Estimate | Standard Error | Z-value | P-value |
| --- | --- | --- | --- | --- |
| Huang et al. 2019 | 0.091 | 0.020 | 4.47 | 7.49e-06 |
| Lamichhane et al. 2018 | 0.094 | 0.020 | 4.59 | 4.30e-06 |
| Mirabelli et al. 2016 | 0.087 | 0.020 | 4.36 | 1.293e-05 |
| Fan et al. 2016 | 0.136 | 0.029 | 4.71 | 2.45e-06 |
| Cortez-Lugo et al. 2015(COPDC) | 0.091 | 0.020 | 4.51 | 6.30e-06 |
| Cortez-Lugo et al. 2015(COPDP) | 0.092 | 0.020 | 4.51 | 6.30e-06 |
| Mar et al. 2004 | 0.098 | 0.020 | 4.71 | 2.46e-06 |
| Marchetti et al. 2023 (R) | 0.090 | 0.020 | 4.53 | 5.70e-06 |
| Aron et al. 2023 | 0.112 | 0.024 | 4.66 | 3.04e-06 |
| Kangas et al. 2023 | 0.071 | 0.019 | 3.60 | 0.0003 |
| Shin et al. 2021 | 0.141 | 0.035 | 4.01 | 6.04e-05 |

**TABLE S 4** PM 2.5 sensitivity analysis model summaries for adults

| Model | Effect Size Estimate | Standard Error | Z-Value | P-Value | ci.lb | ci.ub | tau^2 | I^2 |
| --- | --- | --- | --- | --- | --- | --- | --- | --- |
| Original | 0.0960 | 0.0204 | 4.7036 | <.0001 | 0.0560 | 0.1360 | 0.0016 | 88.88% |
| Overall | 0.0960 | 0.0204 | 4.7036 | <.0001 | 0.0560 | 0.1360 | 0.0016 | 88.88% |
|  |  |  |  |  |  |  |  |  |

**TABLE S 5** Sensitivity analysis of respiratory disease of PM 2.5 for all

| Excluded Study | Effect Size Estimate | Standard Error | Z-value | P-value |
| --- | --- | --- | --- | --- |
| Yu et al. 2020" | 0.23 | 0.05 | 4.00 | 6.2e-05 |
| Fan et al. 2016 | 0.25 | 0.06 | 4.16 | 3.1e-05 |
| Raaschou-Nielsen et al. 2013(LC) | 0.22 | 0.05 | 4.15 | 3.3e-05 |
| Raaschou-Nielsen et al. 2013(A) | 0.21 | 0.05 | 4.10 | 4.0e-05 |
| Kloog et al. 2013 | 0.20 | 0.05 | 4.12 | 3.7e-05 |
| Katanoda et al. 2011 | 0.23 | 0.05 | 4.09 | 4.3e-05 |
| Yan et al. 2022 (CB) | 0.23 | 0.05 | 4.20 | 2.6e-05 |
| Yan et al. 2022 (AT) | 0.23 | 0.05 | 4.17 | 2.9e-05 |
| Yan et al. 2022 (COPD) | 0.25 | 0.05 | 4.67 | 3.0e-06 |
| Yan et al. 2022 (CRD) | 0.23 | 0.05 | 4.19 | 2.6e-05 |
| Zheng et al. 2024(AT) | 0.23 | 0.05 | 4.14 | 3.4e-05 |
| Zheng et al. 2024(W) | 0.22 | 0.05 | 4.06 | 4.7e-05 |
| Zheng et al. 2024(D) | 0.18 | 0.04 | 3.94 | 7.9e-05 |
| Mebrahtu et al. 2023 | 0.24 | 0.06 | 3.57 | 0.0003 |

**TABLE S 6** PM 2.5 sensitivity analysis model summaries for all

| Model | Effect Size Estimate | Standard Error | Z-Value | P-Value | ci.lb | ci.ub | tau^2 | I^2 |
| --- | --- | --- | --- | --- | --- | --- | --- | --- |
| Original | 0.2283 | 0.0531 | 4.3016 | <.0001 | 0.1243 | 0.3324 | 0.0311 | 96.03% |
| Overall | 0.2186 | 0.0532 | 4.1064 | <.0001 | 0.1143 | 0.3229 | 0.0307 | 96.29% |
|  |  |  |  |  |  |  |  |  |

**TABLE S 7** Sensitivity analysis of respiratory disease of PM 10 for children

| Excluded Study | Effect Size Estimate | Standard Error | Z-value | P-value |
| --- | --- | --- | --- | --- |
| Weinmayr et al. 2010(AS) | 0.04 | 0.02 | 1.87 | 0.06 |
| Weinmayr et al. 2010(C) | 0.04 | 0.01 | 3.11 | 0.001 |
| Mar et al. 2004(TB) | 0.03 | 0.01 | 2.39 | 0.016 |
| Mar et al. 2004(C) | 0.02 | 0.008 | 2.39 | 0.016 |
| Mar et al. 2004(SP) | 0.02 | 0.01 | 2.29 | 0.021 |

**TABLE S 8** PM 10 sensitivity analysis model summaries for children

| Model | Effect Size Estimate | Standard Error | Z-Value | P-Value | ci.lb | ci.ub | tau^2 | I^2 |
| --- | --- | --- | --- | --- | --- | --- | --- | --- |
| Original | 0.0308 | 0.0119 | 2.5796 | 0.0099 | 0.0074 | 0.0542 | 0.0003 | 52.98% |
| Overall | 0.0308 | 0.0119 | 2.5796 | 0.0099 | 0.0074 | 0.0542 | 0.0003 | 52.98% |
|  |  |  |  |  |  |  |  |  |

**TABLE S 9** Sensitivity analysis of respiratory disease of PM 10 for adults

**TABLE S 10** PM 10 sensitivity analysis model summaries for adults

| Model | Effect Size Estimate | Standard Error | Z-Value | P-Value | ci.lb | ci.ub | tau^2 | I^2 |
| --- | --- | --- | --- | --- | --- | --- | --- | --- |
| Original | 0.0576 | 0.0325 | 1.7725 | 0.0763 | -0.0061 | 0.1212 | 0.0032 | 64.86% |
| Overall | 0.0576 | 0.0325 | 1.7725 | 0.0763 | -0.0061 | 0.1212 | 0.0032 | 64.86% |
|  |  |  |  |  |  |  |  |  |

| Excluded Study | Effect Size Estimate | Standard Error | Z-value | P-value |
| --- | --- | --- | --- | --- |
| Magzamen et al. 2018 | 0.05 | 0.04 | 1.21 | 0.228 |
| Lamichhane et al. 2018 | 0.05 | 0.03 | 1.62 | 0.108 |
| Mar et al. 2004(W) | 0.07 | 0.04 | 1.85 | 0.068 |
| Mar et al. 2004(TB) | 0.07 | 0.04 | 1.75 | 0.08 |
| Mar et al. 2004(SP) | 0.07 | 0.03 | 1.84 | 0.066 |
| Marchetti et al. 2023 (R) | 0.04 | 0.02 | 1.88 | 0.06 |

**TABLE S 11** Sensitivity analysis of respiratory disease of PM 10 for all

| Excluded Study | Effect Size Estimate | Standard Error | Z-value | P-value |
| --- | --- | --- | --- | --- |
| "Raaschou-Nielsen et al. 2013(LC)" | 0.30 | 0.11 | 2.52 | 0.01 |
| "Raaschou-Nielsen et al. 2013(A)" | 0.26 | 0.10 | 2.40 | 0.01 |
| "Analitis et al. 2006" | 0.33 | 0.12 | 2.58 | 0.009 |
| "Zheng et al. 2024(AT)" | 0.28 | 0.11 | 2.48 | 0.01 |
| "Zheng et al. 2024(W)" | 0.27 | 0.11 | 2.40 | 0.01 |
| "Zheng et al. 2024(D)" | 0.16 | 0.06 | 2.63 | 0.008 |
| "Mebrahtu et al. 2023" | 0.32 | 0.14 | 2.30 | 0.02 |

**TABLE S 12** PM 10 sensitivity analysis model summaries for all

| Model | Effect Size Estimate | Standard Error | Z-Value | P-Value | ci.lb | ci.ub | tau^2 | I^2 |
| --- | --- | --- | --- | --- | --- | --- | --- | --- |
| Original | 0.2798 | 0.1029 | 2.7194 | 0.0065 | 0.0781 | 0.4815 | 0.0667 | 95.96% |
| Overall | 0.2798 | 0.1029 | 2.7194 | 0.0065 | 0.0781 | 0.4815 | 0.0667 | 95.96% |
|  |  |  |  |  |  |  |  |  |

**TABLE S 13** Sensitivity analysis of respiratory disease of NO2 for children

| Excluded Study | Effect Size Estimate | Standard Error | Z-value | P-value |
| --- | --- | --- | --- | --- |
| Hasunuma et al. 2016 | 0.11 | 0.037 | 3.004 | 0.002 |
| Belanger et al. 2013(AS) | 0.10 | 0.03 | 2.985 | 0.002 |
| Belanger et al. 2013(W) | 0.10 | 0.03 | 2.95 | 0.003 |
| Belanger et al. 2013(NSA) | 0.10 | 0.03 | 2.92 | 0.003 |
| Belanger et al. 2013(MUA) | 0.08 | 0.02 | 3.01 | 0.002 |
| Takenoue et al. 2012(AD) | 0.11 | 0.04 | 2.81 | 0.004 |
| Takenoue et al. 2012(W) | 0.18 | 0.05 | 3.23 | 0.001 |
| Weinmayr et al. 2010 | 0.15 | 0.04 | 3.16 | 0.001 |
| Migliaretti and Cavallo 2004 | 0.17 | 0.05 | 3.22 | 0.001 |

**TABLE S 14** NO2 sensitivity analysis model summaries for children

| Model | Effect Size Estimate | Standard Error | Z-Value | P-Value | ci.lb | ci.ub | tau^2 | I^2 |
| --- | --- | --- | --- | --- | --- | --- | --- | --- |
| Original | 0.1137 | 0.0360 | 3.1555 | 0.0016 | 0.0431 | 0.1843 | 0.0048 | 75.78% |
| Overall | 0.0911 | 0.0323 | 2.8194 | 0.0048 | 0.0278 | 0.1544 | 0.0036 | 75.37% |
|  |  |  |  |  |  |  |  |  |

**TABLE S 15** Sensitivity analysis of respiratory disease of NO2 for adults

| Excluded Study | Effect Size Estimate | Standard Error | Z-value | P-value |
| --- | --- | --- | --- | --- |
| Magzamen et al. 2018 | 0.14 | 0.04 | 3.28 | 0.001 |
| Lamichhane et al. 2018 | 0.10 | 0.02 | 3.84 | 0.0001 |
| De Marco et al. 2002(AA) | 0.11 | 0.02 | 3.85 | 0.0001 |
| De Marco et al. 2002(CT) | 0.11 | 0.02 | 3.85 | 0.0001 |
| De Marco et al. 2002(W) | 0.11 | 0.02 | 3.89 | 0.0001 |
| Shin et al. 2021 | 0.14 | 0.04 | 3.57 | 0.0003 |
| Marchetti et al. 2023 (COPD) | 0.09 | 0.02 | 3.60 | 0.0003 |
| Kwon et al. 2024 | 0.07 | 0.01 | 3.69 | 0.0002 |

**TABLE S 16**  NO2 sensitivity analysis model summaries for adults

| Model | Effect Size Estimate | Standard Error | Z-Value | P-Value | ci.lb | ci.ub | tau^2 | I^2 |
| --- | --- | --- | --- | --- | --- | --- | --- | --- |
| Original | 0.1110 | 0.0272 | 4.0872 | <.0001 | 0.0578 | 0.1642 | 0.0028 | 72.08% |
| Overall | 0.1110 | 0.0272 | 4.0872 | <.0001 | 0.0578 | 0.1642 | 0.0028 | 72.08% |
|  |  |  |  |  |  |  |  |  |

**TABLE S 17** Sensitivity analysis of respiratory disease of NO2 for all

| Excluded Study | Effect Size Estimate | Standard Error | Z-value | P-value |
| --- | --- | --- | --- | --- |
| Ghozikali et al. 2016 | 0.09 | 0.02 | 3.16 | 0.001 |
| Li et al. 2016a | 0.08 | 0.03 | 2.23 | 0.02 |
| Katanoda et al. 2011 | 0.04 | 0.01 | 2.67 | 0.007 |
| Sunyer et al. 2002 | 0.06 | 0.01 | 3.33 | 0.0008 |
| Mebrahtu et al. 2023 | 0.05 | 0.02 | 2.63 | 0.008 |

| **TABLE S 18**  NO2 sensitivity analysis model summaries for all   \| Model \| Effect Size Estimate \| Standard Error \| Z-Value \| P-Value \| ci.lb \| ci.ub \| tau^2 \| I^2 \| \| --- \| --- \| --- \| --- \| --- \| --- \| --- \| --- \| --- \| \| Original \| 0.0614 \| 0.0180 \| 3.4100 \| 0.0006 \| 0.0261 \| 0.0966 \| 0.0010 \| 89.05% \| \| Overall \| 0.0614 \| 0.0180 \| 3.4100 \| 0.0006 \| 0.0261 \| 0.0966 \| 0.0010 \| 89.05% \| \|  \|  \|  \|  \|  \|  \|  \|  \|  \|     **TABLE S 19** Sensitivity analysis of respiratory disease of O3 for children   \| Excluded Study \| Effect Size Estimate \| Standard Error \| Z-value \| P-value \| \| --- \| --- \| --- \| --- \| --- \| \| Pepper et al. 2020 \| 0.01 \| 0.007 \| 2.57 \| 0.01 \| \| Gent et al. 2003(CTOH) \| 0.02 \| 0.007 \| 2.79 \| 0.005 \| \| Gent et al. 2003(SOBOH) \| 0.02 \| 0.007 \| 2.79 \| 0.005 \| \| Gent et al. 2003(CTEH) \| 0.02 \| 0.007 \| 2.74 \| 0.006 \| \| Gent et al. 2003(SOBEH) \| 0.02 \| 0.007 \| 2.81 \| 0.004 \| \| Stowell et al. 2024(AL) \| 0.01 \| 0.007 \| 2.11 \| 0.03 \| \| Stowell et al. 2024(AT) \| 0.03 \| 0.009 \| 3.22 \| 0.001 \| \| Stowell et al. 2024(RD) \| 0.04 \| "0.012 \| 3.59 \| 0.0003 \| \| Stowell et al. 2024(RI) \| 0.04 \| 0.012 \| 3.59 \| 0.0003 \| |
| --- | --- | --- | --- | --- | --- | --- | --- | --- | --- | --- | --- | --- | --- | --- | --- | --- | --- | --- | --- | --- | --- | --- | --- | --- | --- | --- | --- | --- | --- | --- | --- | --- | --- | --- | --- | --- | --- | --- | --- | --- | --- | --- | --- | --- | --- | --- | --- | --- | --- | --- | --- | --- | --- | --- | --- | --- | --- | --- | --- | --- | --- | --- | --- | --- | --- | --- | --- | --- | --- | --- | --- | --- | --- | --- | --- | --- | --- | --- | --- | --- | --- | --- | --- | --- | --- | --- |
|  |
|  |

**TABLE S 20**  O3 sensitivity analysis model summaries for children

| Model | Effect Size Estimate | Standard Error | Z-Value | P-Value | ci.lb | ci.ub | tau^2 | I^2 |
| --- | --- | --- | --- | --- | --- | --- | --- | --- |
| Original | 0.0244 | 0.0080 | 3.0729 | 0.0021 | 0.0089 | 0.0400 | 0.0003 | 88.06% |
| Overall | 0.0244 | 0.0080 | 3.0729 | 0.0021 | 0.0089 | 0.0400 | 0.0003 | 88.06% |
|  |  |  |  |  |  |  |  |  |

**TABLE S 21** Sensitivity analysis of respiratory disease of O3 for adults

| Excluded Study | Effect Size Estimate | Standard Error | Z-value | P-value |
| --- | --- | --- | --- | --- |
| Pepper et al. 2020 | 0.17 | 0.06 | 2.83 | 0.004 |
| Day et al. 2017 | 0.15 | 0.05 | 3.00 | 0.002 |
| Silverman and Ito 2010 | 0.14 | 0.05 | 2.67 | 0.007 |
| Shin et al. 2021 | 0.18 | 0.02 | 7.09 | 1.2e-12 |
| Zhang et al. 2024 | 0.16 | 0.05 | 2.87 | 0.003 |

**TABLE S 22**  O3 sensitivity analysis model summaries for adults

| Model | Effect Size Estimate | Standard Error | Z-Value | P-Value | ci.lb | ci.ub | tau^2 | I^2 |
| --- | --- | --- | --- | --- | --- | --- | --- | --- |
| Original | 0.1672 | 0.0498 | 3.3576 | 0.0008 | 0.0696 | 0.2648 | 0.0094 | 96.37% |
| Overall | 0.1672 | 0.0498 | 3.3576 | 0.0008 | 0.0696 | 0.2648 | 0.0094 | 96.37% |
|  |  |  |  |  |  |  |  |  |

**TABLE S 23** Sensitivity analysis of respiratory disease of O3 for all

| Excluded Study | Effect Size Estimate | Standard Error | Z-value | P-value |
| --- | --- | --- | --- | --- |
| Khaniabadi et al. 2017(CM) | 0.24 | 0.08 | 2.98 | 0.002 |
| Khaniabadi et al. 2017(COPDH) | 0.27 | 0.09 | 2.89 | 0.003 |
| Ghozikali et al. 2016 | 0.31 | 0.11 | 2.74 | 0.006 |
| Yang et al. 2024 | 0.32 | 0.10 | 3.22 | 0.001 |
| Xing et al. 2024 | 0.16 | 0.04 | 3.40 | 0.0006 |

**TABLE S 24** O3 sensitivity analysis model summaries for all

| Model | Effect Size Estimate | Standard Error | Z-Value | P-Value | ci.lb | ci.ub | tau^2 | I^2 |
| --- | --- | --- | --- | --- | --- | --- | --- | --- |
| Original | 0.2643 | 0.0757 | 3.4902 | 0.0005 | 0.1159 | 0.4126 | 0.0240 | 89.89% |
| Overall | 0.2643 | 0.0757 | 3.4902 | 0.0005 | 0.1159 | 0.4126 | 0.0240 | 89.89% |
|  |  |  |  |  |  |  |  |  |

**TABLE S 25** Sensitivity analysis of respiratory disease of SO2 for children

| Excluded Study | Effect Size Estimate | Standard Error | Z-value | P-value |
| --- | --- | --- | --- | --- |
| Greenberg et al. 2016 | 0.30 | 0.19 | 1.55 | 0.12 |
| Smargiassi et al. 2009(AEDV) | 0.32 | 0.15 | 2.05 | 0.04 |
| Smargiassi et al. 2009(AH) | 0.19 | 0.04 | 4.23 | 2.2e-05 |

**TABLE S 26** SO2 sensitivity analysis model summaries for children

| Model | Effect Size Estimate | Standard Error | Z-Value | P-Value | ci.lb | ci.ub | tau^2 | I^2 |
| --- | --- | --- | --- | --- | --- | --- | --- | --- |
| Original | 0.2232 | 0.0684 | 3.2648 | 0.0011 | 0.0892 | 0.3571 | 0.0059 | 41.86% |
| Overall | 0.2232 | 0.0684 | 3.2648 | 0.0011 | 0.0892 | 0.3571 | 0.0059 | 41.86% |
|  |  |  |  |  |  |  |  |  |

**TABLE S 27** Sensitivity analysis of respiratory disease of SO2 for adults

| Excluded Study | Effect Size Estimate | Standard Error | Z-value | P-value |
| --- | --- | --- | --- | --- |
| Mercan et al. 2020(AH) | 0.09 | 0.04 | 2.04 | 0.04 |
| Mercan et al. 2020(COPDH) | 0.10 | 0.05 | 1.91 | 0.05 |
| Li et al. 2016b | 0.12 | 0.04 | 2.75 | 0.005 |
| Kan et al. 2010 | 0.15 | 0.01 | 9.14 | 6.1e-20" |

**TABLE S 28** SO2 sensitivity analysis model summaries for adults

| Model | Effect Size Estimate | Standard Error | Z-Value | P-Value | ci.lb | ci.ub | tau^2 | I^2 |
| --- | --- | --- | --- | --- | --- | --- | --- | --- |
| Original | 0.1171 | 0.0389 | 3.0104 | 0.0026 | 0.0409 | 0.1933 | 0.0058 | 97.51% |
| Overall | 0.1171 | 0.0389 | 3.0104 | 0.0026 | 0.0409 | 0.1933 | 0.0058 | 97.51% |
|  |  |  |  |  |  |  |  |  |

**TABLE S 29** Sensitivity analysis of respiratory disease of SO2 for all

| Excluded Study | Effect Size Estimate | Standard Error | Z-value | P-value |
| --- | --- | --- | --- | --- |
| Ghozikali et al. 2016 | 0.11 | 0.09 | 1.14 | "0.25 |
| Li et al. 2016a | 0.10 | 0.10 | 0.96 | "0.33 |
| Katanoda et al. 2011 | 0.01 | 0.008 | 1.75 | "0.07 |

**TABLE S 30** SO2 sensitivity analysis model summaries for all

| Model | Effect Size Estimate | Standard Error | Z-Value | P-Value | ci.lb | ci.ub | tau^2 | I^2 |
| --- | --- | --- | --- | --- | --- | --- | --- | --- |
| Original | 0.0315 | 0.0213 | 1.4763 | 0.1399 | -0.0103 | 0.0733 | 0.0008 | 75.68% |
| Overall | 0.0315 | 0.0213 | 1.4763 | 0.1399 | -0.0103 | 0.0733 | 0.0008 | 75.68% |

**Table S 31** Sensitivity analysis of respiratory disease of trace metal for children

| Excluded Study | Effect Size Estimate | Standard Error | Z-value | P-value |
| --- | --- | --- | --- | --- |
| Wu et al. 2019(RAA)(Pb) | 0.01 | 0.006 | 2.49 | 0.01 |
| Wu et al. 2019(W)(Pb) | 0.018 | 0.007 | 2.42 | 0.01 |
| Pollitt et al. 2016(AIDA)(Al) | 0.02 | 0.009 | 2.35 | 0.01 |
| Pollitt et al. 2016(AIDA)(Fe) | 0.02 | 0.009 | 2.57 | 0.01 |
| Pollitt et al. 2016(AIDA)(Mg) | 0.02 | 0.009 | 2.50 | 0.01 |
| Pollitt et al. 2016(AIDA)(S) | 0.02 | 0.009 | 2.42 | 0.01 |
| Pollitt et al. 2016(AIDA)(Ni) | 0.024 | 0.009 | 2.57 | 0.01 |
| Pollitt et al. 2016(AIDA)(V) | 0.021 | 0.008 | 2.42 | 0.01 |
| Pollitt et al. 2016(AIDA)(Cr) | 0.025 | 0.009 | 2.56 | 0.01 |
| Pollitt et al. 2016(AIDA)(As) | 0.022 | 0.008 | 2.49 | 0.01 |
| Pollitt et al. 2016(AIDA)(Mn) | 0.025 | 0.008 | 2.87 | 0.004 |
| Pollitt et al. 2016(AIDA)(Ba) | 0.017 | 0.007 | 2.19 | 0.02 |
| Pollitt et al. 2016(AIDA)(Cu) | 0.024 | 0.010 | 2.40 | 0.01 |
| Pollitt et al. 2016(AIDA)(Sb) | 0.022 | 0.008 | 2.56 | 0.01 |
| Pollitt et al. 2016(AIDA)(Zn) | 0.025 | 0.008 | 2.87 | 0.004 |

**Table S 32**  Trace metal sensitivity analysis model summaries for children

| Model | Effect Size Estimate | Standard Error | Z-Value | P-Value | ci.lb | ci.ub | tau^2 | I^2 |
| --- | --- | --- | --- | --- | --- | --- | --- | --- |
| Original | 0.0220 | 0.0085 | 2.5891 | 0.0096 | 0.0054 | 0.0387 | 0.0004 | 41.84% |
| Overall | 0.0220 | 0.0085 | 2.5891 | 0.0096 | 0.0054 | 0.0387 | 0.0004 | 41.84% |

**TABLE S 33** Sensitivity analysis of respiratory disease of trace metal for adults

| Excluded Study | Effect Size Estimate | Standard Error | Z-value | P-value |
| --- | --- | --- | --- | --- |
| Wang et al. 2023(LF)(As) | 0.009 | 0.41 | 0.02 | 0.98 |
| Wang et al. 2023(B)(As) | 0.33 | 0.15 | 2.18 | 0.02 |

**TABLE S 34** Trace metal sensitivity analysis model summaries for adults

| Model | Effect Size Estimate | Standard Error | Z-Value | P-Value | ci.lb | ci.ub | tau^2 | I^2 |
| --- | --- | --- | --- | --- | --- | --- | --- | --- |
| Original | 0.2967 | 0.1441 | 2.0595 | 0.0394 | 0.0143 | 0.5791 | 0 | 0.00% |
| Overall | 0.3365 | 0.1537 | 2.1887 | 0.0286 | 0.0352 | 0.6378 | 0 | 0.00% |

**TABLE S 35** Sensitivity analysis of respiratory disease of trace metal for all

| Excluded Study | Effect Size Estimate | Standard Error | Z-value | P-value |
| --- | --- | --- | --- | --- |
| Mao et al. 2018(AS)(Cu) | 0.60 | 0.36 | 1.65 | 0.09 |
| Mao et al. 2018(AS)(Fe) | 0.44 | 0.35 | 1.27 | 0.20 |
| Yu et al. 2024(T)(Zn) | 0.77 | 0.18 | 4.20 | 2.6e-05 |
| Yu et al. 2024(E)(Cu) | 0.35 | 0.32 | 1.10 | 0.27 |
| Yu et al. 2024(COPD)(Cu) | 0.47 | 0.38 | 1.24 | 0.21 |
| Yu et al. 2024(COPD)(Zn) | 0.50 | 0.39 | 1.26 | 0.20 |

| Model | Effect Size Estimate | Standard Error | Z-Value | P-Value | ci.lb | ci.ub | tau^2 | I^2 |
| --- | --- | --- | --- | --- | --- | --- | --- | --- |
| Original | 0.5215 | 0.3208 | 1.6257 | 0.1040 | -0.1072 | 1.1503 | 0.4447 | 76.59% |
| Overall | 0.0794 | 0.5463 | 0.1453 | 0.8845 | -0.9913 | 1.1501 | 0.5242 | 87.69% |

**TABLE S 36** Trace metal sensitivity analysis model summaries for all

**TABLE S 37** Bias analysis of the studies included in this review using Newcastle–Ottawa scale (NOS)

| First Author | Year | Selection | | | | Comparability | | Outcome | | | Total |
| --- | --- | --- | --- | --- | --- | --- | --- | --- | --- | --- | --- |
|  |  | Representativeness | Selection | Ascertainment | Demonstration | Study controls for an important factor | Study controls for any additional factor | Assessment | Duration of follow-up | Adequacy |  |
| De Marco | 2002 | * | * | * | * | * | * | * | * | * | 9 |
| Sunyer | 2002 | * | Na | * | * | * | * | * | * | * | 8 |
| Gent | 2003 | * | Na | * | Na | * | * | * | * | * | 7 |
| Mar | 2004 | * | Na | * | Na | * | * | * | * | * | 7 |
| Migliaretti | 2004 | * | * | * | * | * | * | * | * | * | 9 |
| Analitis | 2006 | * | Na | * | Na | * | * | * | * | * | 7 |
| Brauer | 2007 | * | * | * | * | * | * | * | * | * | 9 |
| Smargiassi | 2009 | * | Na | * | * | * | * | * | * | * | 8 |
| Kan | 2010 | * | Na | * | Na | * | * | * | * | * | 7 |
| Silverman | 2010 | * | Na | * | Na | * | * | * | * | * | 7 |
| Weinmayr | 2010 | * | Na | * | * | * | * | * | * | * | 8 |
| Katanoda | 2011 | * | Na | * | * | * | * | * | * | * | 8 |
| Takenou | 2012 | * | Na | * | * | * | * | * | * | * | 8 |
| Belanger | 2013 | * | Na | * | * | * | * | * | * | * | 8 |
| Kloog | 2013 | * | Na | * | Na | * | * | * | * | * | 7 |
| Raaschou-Nielsen | 2013 | * | Na | * | * | * | * | * | * | * | 8 |
| Cortez-Lugo | 2015 | * | * | * | * | * | * | * | * | * | 9 |
| Fan | 2016 | * | Na | * | * | * | * | * | * | * | 8 |
| Ghozikali | 2016 | * | Na | * | Na | * | * | * | * | * | 7 |
| Greenberg | 2016 | * | Na | * | Na | * | * | * | * | * | 7 |
| Hasunuma | 2016 | * | * | * | * | * | * | * | * | * | 9 |
| Mirabelli | 2016 | * | Na | * | Na | * | * | * | * | * | 7 |
| Pollitt | 2016 | * | Na | * | Na | * | * | * | * | * | 7 |
| Li | 2016a | * | Na | * | * | * | * | * | * | Na | 7 |
| Li | 2016b | * | Na | * | * | * | * | * | * | Na | 7 |
| Day | 2017 | * | Na | * | * | * | * | * | * | * | 8 |
| Khaniabadi | 2017 | * | Na | * | * | * | * | * | * | * | 8 |
| Lamichhane | 2018 | * | Na | * | * | * | * | * | Na | Na | 6 |
| Magzamen | 2018 | * | Na | * | Na | * | * | * | * | * | 7 |
| Mao | 2018 | * | * | * | * | * | * | * | Na | Na | 7 |
| Hansel | 2019 | * | Na | * | Na | * | * | * | * | * | 7 |
| Huang | 2019 | * | Na | * | * | * | * | * | * | * | 8 |
| Wu | 2019 | * | * | * | Na | * | * | * | Na | * | 7 |
| Mercan | 2020 | * | Na | * | Na | * | * | * | * | * | 7 |
| Pepper | 2020 | * | Na | * | Na | * | * | * | * | * | 7 |
| Yu | 2020 | * | Na | * | Na | * | * | * | * | * | 7 |
| Shin | 2021 | * | Na | * | * | * | * | * | * | * | 8 |
| Yan | 2022 | * | Na | * | * | * | * | * | * | Na | 7 |
| Mebrahtu | 2023 | * | * | * | * | * | * | * | * | * | 9 |
| Aron | 2023 | * | Na | * | * | * | * | * | * | * | 8 |
| Kangas | 2023 | * | * | * | * | * | * | * | * | * | 9 |
| Marchetti | 2023 | * | * | * | Na | * | * | * | Na | Na | 6 |
| Wang | 2023 | * | * | * | * | * | * | * | * | * | 9 |
| Yang | 2024 | * | * | * | * | * | * | * | * | * | 9 |
| Stowell | 2024 | * | Na | * | * | * | * | * | * | Na | 7 |
| Yu | 2024 | * | * | * | * | * | * | * | * | * | 9 |
| Kwon | 2024 | * | * | * | * | * | * | * | * | * | 9 |
| Zhang | 2024 | * | * | * | * | * | * | * | * | Na | 8 |
| Zheng | 2024 | * | * | * | * | * | * | * | * | * | 9 |
| Xing | 2024 | * | * | * | * | * | * | * | Na | Na | 7 |

**FIGURE S 1** Funnel plot of the odds ratio of developing respiratory disease, according to the standard error for PM 2.5.

**FIGURE S 2** Funnel plot of the odds ratio of developing respiratory disease, according to the standard error for PM 10

**FIGURE S 3** Funnel plot of the odds ratio of developing respiratory disease, according to the standard error for NO2

**FIGURE S 4**  Funnel plot of the odds ratio of developing respiratory disease, according to the standard error for O3

**FIGURE S 5** Funnel plot of the odds ratio of developing respiratory disease, according to the standard error for SO2

**FIGURE S 6**  Funnel plot of the odds ratio of developing respiratory disease, according to the standard error for Trace metals
